# Spatial distribution and its limiting environmental factors of native orchid species diversity in the Beipan River Basin of Guizhou Province, China

**DOI:** 10.1002/ece3.9470

**Published:** 2022-11-14

**Authors:** Chao Ye, Mingtai An, Jinzhu Shi, Feng Liu, Yang Zhang

**Affiliations:** ^1^ College of Forestry Guizhou University Guiyang China

**Keywords:** Beipan river, distribution pattern, environmental factors, orchidaceae

## Abstract

To understand the distribution of biodiversity and its determinants, particularly that of ecologically sensitive ones, has long been intriguing to the science community and will help formulate conservation strategies under future climate changes. To this end, we conducted extensive field surveys on the distribution of orchid flora in the Beipan River Basin in Guizhou Province, which is one of the biodiversity conservation priorities in China. The data we acquired, together with those published previously, were converted into orchid species richness for each of the 3 km × 3 km grid cells covering the study region. Redundancy analysis (RDA) and geographically weighted regression (GWR) were then applied to determine which of the 30 environmental factors are potentially critical for the spatial distribution of orchid flora we have observed. Despite a moderate spatial extent, we found that the Beipan River Basin harbors about 249 native orchid species belonging to 74 genera, equivalent to 14.5% of orchid flora of China. Orchid species richness in this area follows a descending gradient from the southeast to the northwest, 70.41% of its variation among grid cells can be explained by environmental factors and spatial variables, and spatial variables accounted for 63.90% of the spatial variation of orchid distribution, indicating that spatial variables played a dominant role in the distribution of wild Orchidaceae species richness. In addition, the main environmental driver is the mean temperature of the wettest quarter. Our study provides a good example for revealing the main drivers of orchid distribution characteristics and has a certain reference value for the development of orchid conservation strategies.

## Graphical abstract

Despite a moderate spatial extent, we found that the Beipan River Basin harbors about 249 native orchid species belonging to 74 genera, equivalent to 14.5% of orchid in China. Orchid species richness in this area follows a descending gradient from the southeast to the northwest, 70.41% of its variation among grid cells can be explained by environmental factors and spatial variables, and the main environmental driver is mean temperature of the wettest quarter.

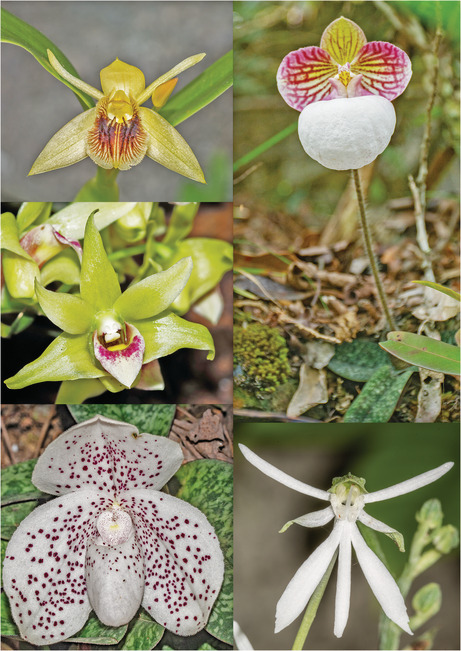

## 1 INTRODUCTION

With about 28,000 species and 736 genera recognized to date (Christenhusz & Byng, 2016), orchid is among the most evolved, and diverse plant families. Species in this family are characterized by the highly specialized structure of their flowers (Kong et al., 2012), many of which are showy. Unfortunately, over‐collection for their medicinal and ornamental value, as well as their inherent ecological sensitivities (Yin et al., 2018), has rendered many orchid species threatened by extinction (Swarts & Dixon, 2009). In addition, because they are widely distributed and occur in a wide spectrum of habitats, this makes orchids an important group for biological conservation and an ideal group for exploring species distribution models (Tsiftsis et al., 2019).

Southwest China is one of the world‐renowned biodiversity hotspots (Myers et al., 2000). This region is also extremely rich in orchid diversity (Dixon et al., 2003). Located in southwest China, Guizhou Province is characterized by well‐developed karst landforms, which cover 61.9% of its total land area. The Beipan River Basin is located in the southwest of Guizhou Province. Despite its moderate spatial extent, the Beipan River Basin is a major intersection of the southwest and southeast Asian monsoon and is also the hinterland with the most obvious and developed karst geological structure in Guizhou Province. Prominent environmental heterogeneity and desirable vapor availability in the Beipan River Basin produce favorable habitats for orchid species. It is, therefore, an ideal place for investigating the spatial distribution of orchid flora and unraveling dominant environmental factors underlying such distribution.

The present distribution of biodiversity results from long‐term interaction among organisms, or between organisms and the abiotic environment (Sterner et al., 1986). Because organisms vary in their responses to these biotic or abiotic interactions, different patterns of distribution may arise. Since the 18th century, dozens of hypotheses have been purposed to explain the spatial distribution of biodiversity, such as the water‐energy dynamic hypothesis (O'Brien, 2006), ambient energy hypothesis (Wright, 1983), and habitat heterogeneity hypothesis (MacArthur & MacArthur, 1961), and new hypotheses are emerging (Wang et al., 2012). In general, these hypotheses pertain to two broad categories of factors, i.e., present environmental conditions and historical contingency. Environmental conditions are the most important determinants of the distribution of biodiversity on Earth, among which water, energy, and habitat heterogeneity are particularly decisive for plant species richness (Hawkins & Porter, 2010; Wang et al., 2018). Moreover, spatial variables are also important factors influencing the distribution of plant diversity (La et al., 2016), and should be taken into account by relevant studies.

To date, studies on the spatial distribution of orchids, as well as underlying drivers, are relatively scant. Such studies are often based on specimens or floras (Acharya et al., 2011; Phillips et al., 2011), resulting in potential bias due to incomplete species inventories or even incorrect taxon identification. Therefore, it is of great significance to analyze the distribution patterns of orchid species in the study area based on field survey data and to determine the factors underlying such observed patterns of orchid diversity.

## 2 METHODS

### 2.1 Species survey

**FIGURE 1:**
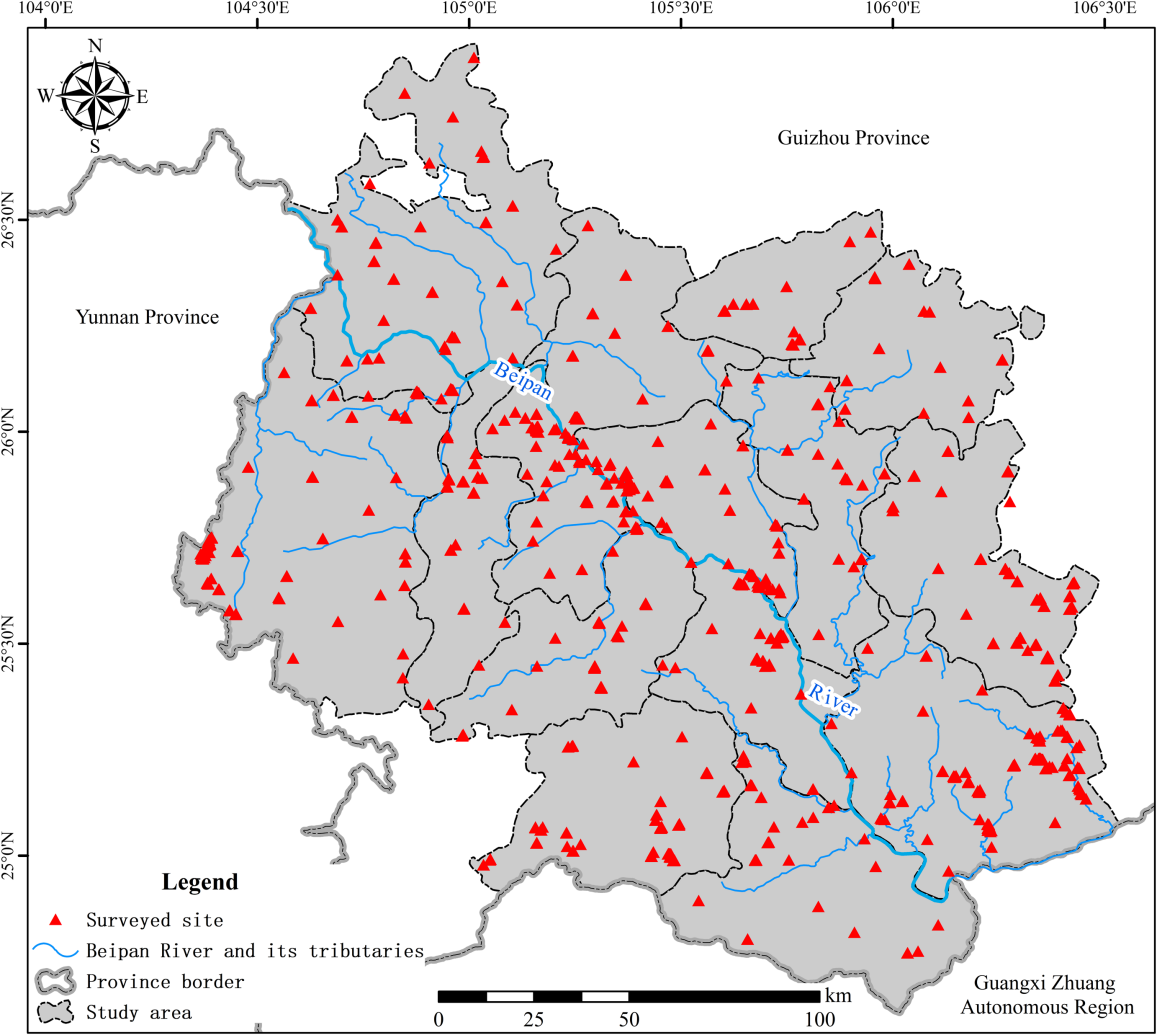
Schematic diagram of investigation sites for Orchidaceae

Orchidaceae spatial distribution data used in this study were derived from a comprehensive dataset that included both field‐based species distribution records and those available in published literatures and floras. Intensive field survey covered the entire study area (Figure 1) and was particularly concentrated in places where historical investigations indicated high orchid diversity, as well as where field survey had not been conducted adequately. We established transects with a length of at least 100 m and set up quadrats (5 m * 5 m) on both sides of each transect. A minimal distance of 10 m was kept between any neighboring quadrats. For each quadrat, we recorded the GPS location, elevation, slope, aspect, orchid species composition (including abundance of each species and abundance of breeding individuals of each species), characteristics of local vegetation (e.g., foundational species), characteristics of local abiotic environments (e.g., type of soil or bedrock), and level of anthropogenic disturbance.

### 2.2 Environmental variables

To identify environmental variables that can explain the observed spatial distribution of native orchids in the Beipan River Basin, we first downloaded a total of 30 environmental factors categorized into four types: energy, water, habitat heterogeneity, and human activities, covering most of those known to have large impacts on the distribution of terrestrial plant diversity.

#### 2.2.1 Energy variables

Altogether 13 energy variables were selected, including mean annual temperature (MAT), mean diurnal range (MDR), isothermality (ISO), temperature seasonality (TS), maximal temperature of the warmest month (MTWM), minimal temperature of the coldest month (MTCM), temperature annual range (TAR), mean temperature of the wettest quarter (MTWetQ), mean temperature of the driest quarter (MTDQ), mean temperature of the coldest quarter (MTCQ), mean temperature of the warmest quarter (MTWarmQ), and potential evapo‐transpiration (PET). All these twelve variables, except PET (from International Agricultural Database, https://cgiarcsi.community) (Zomer et al., 2008), were downloaded from the Worldclim Database (http://www.worldclim.org) and with the resolution of 30 arc seconds (Fick & Hijmans, 2017). Additionally, water deficit (WD) was computed as WD = PET‐MAP (Wang et al., 2018).

#### 2.2.2 Water variables

We selected 10 water variables, including mean annual precipitation (MAP), precipitation of the wettest month (PWM), precipitation of the driest month (PDM), precipitation seasonality (PS), precipitation of the wettest quarter (PWetQ), precipitation of the driest quarter (PDQ), precipitation of the warmest quarter (PWarmQ), precipitation of the coldest quarter (PCQ), and actual evapo‐transpiration (AET). All these nine variables, except AET (Trabucco & Zomer, 2019), were downloaded from the Wordclim Database (http://www. worldclim.org) and with the resolution of 30 arc seconds (Fick & Hijmans, 2017). Additionally, moisture index (MI) was computed as MI = (MAP/PET‐1) × 100 (Thornthwaite, 1948).

#### 2.2.3 Habitat heterogeneity variables

We selected 4 variables that quantify habitat heterogeneity, including elevational range (ER), the number of vegetation formations (NVF) (Ran et al., 2012), and ranges of MAT and MAP (RMAT and RMAP, respectively) for each grid. ER was extracted from a 12.5 m digital elevation model (DEM) (https://search.asf.alaska.edu/), and NVF with the resolution of 1 km^2^ from the Center for Resources and Environmental Science and Data, Chinese Academy of Sciences (http://resdc.cn/).

#### 2.2.4 Human activities variables

We selected 3 variables as proxies of human activity intensity, including human population density (HPD) in 2017 with the resolution of 30 arc seconds downloaded from the Global Demographic Dynamics Statistical Analysis Database (https://landscan.ornl.gov) (Rose et al., 2018), gross domestic product (GDP) in 2015 with the resolution of 1 km^2^ from the Center for Resources and Environmental Science and Data, Chinese Academy of Sciences (http://resdc.cn/) (Xu, 2017), and the area of cropland (AOC) in 2015 with the resolution of 1 km^2^ from the National Qinghai‐Tibet Plateau Scientific Data Center (http://data.tpdc.ac.cn/zh‐hans/) (Chinese Academy of Sciences Resource and Environmental Science Data Center, 2019).

To keep the spatial resolution consistent, the DEM data are resampled to 1 km * 1 km.

### 2.3 Statistical analysis

**FIGURE 2:**
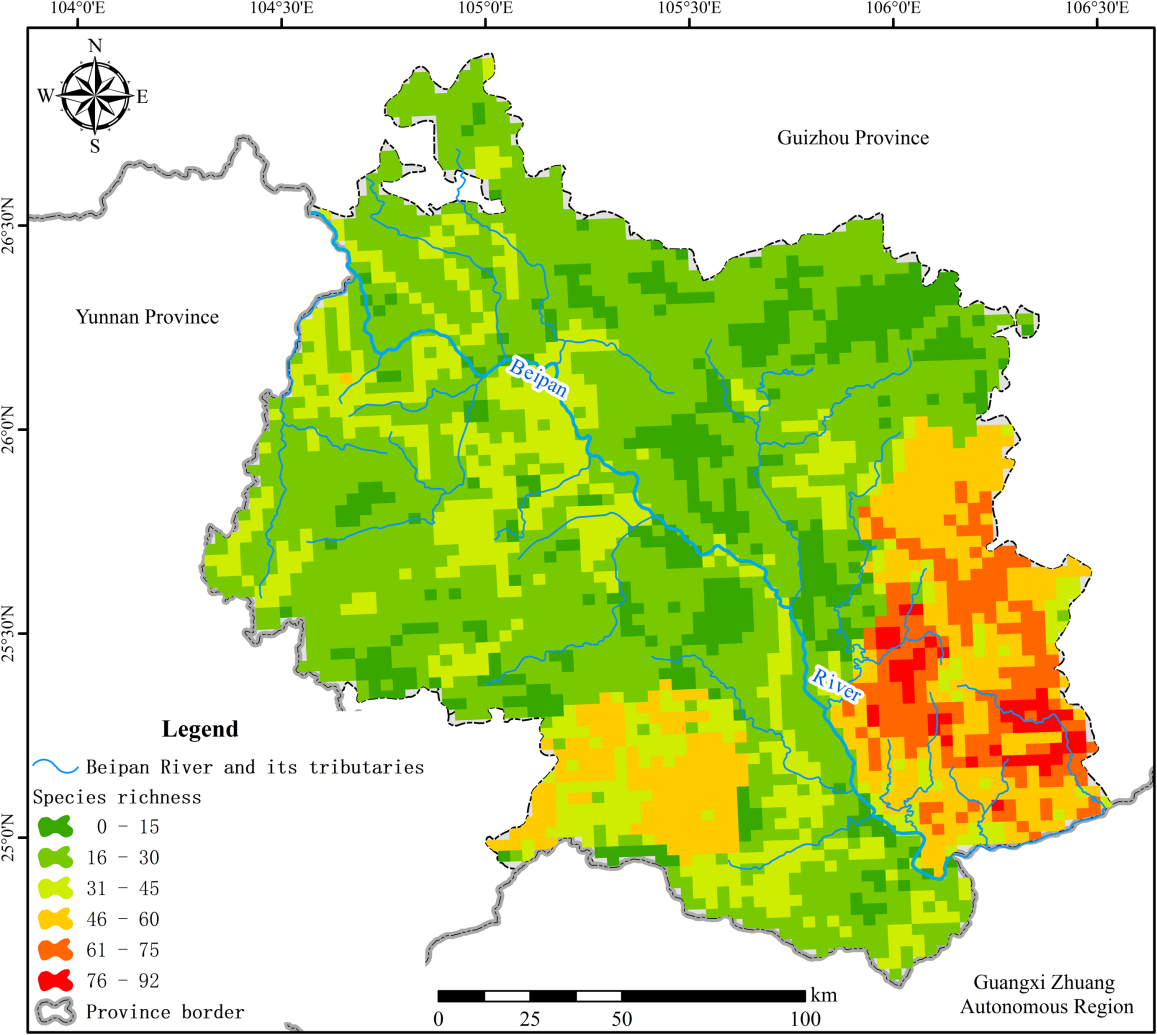
The gradient map of orchid species richness

The study area was divided into 3 km × 3 km grid cells, and the grid cells at the boundaries with more than 1/3 of its area falling out of the study area were excluded, after which 3499 grid cells were retained. Values of energy, water, habitat heterogeneity, and human activity variables for each grid cell was computed as the mean value characterizing the spatial area covered by that grid cell. Then, we also computed orchid species richness for each grid cell. Because it is nearly impossible, within our capacity, to carry out an extensive field survey in each grid cell, this was done in two steps. First, an inventory of orchid species, including the elevational range of each species, was made for each county within the study area, based on our field‐collected data and those available in floras and published literatures. Then, the orchid species richness of each grid cell was computed as the number of orchid species present in the county that the grid cell falls in, and filtered by the elevational range of each species. Note that when a grid cell falls at the adjoining area of >1 counties, it is assumed to fall within the county, which contains the largest proportion of its area (Figure 2).

#### 2.3.1 Linear regression

Linear regressions were made in R3.6.3 (https://www.R‐project.org/) (R Core Team, 2019) to determine the relationship between orchid species richness and each of the environmental variables (including longitude, latitude, and altitude).

#### 2.3.2 Redundancy analysis

Based on the geographical coordinates of the centroid of each grid, a distance‐based Moran's eigenvector map (dbMEM) is generated to create spatial variables, and a two‐step procedure is used to forward the selection of spatial variables and environmental variables. We used these selected variables to explain the spatial distribution of orchid species richness in the study area by using redundancy analysis (RDA) and variance partitioning (Borcard et al., 1992; Smith & Lundholm, 2010), so as to identify the contribution of these variables in shaping the spatial pattern of orchid diversity we have observed.

#### 2.3.3 Principal component analysis

In order to deal with multicollinearity among environmental variables, principal component analysis (PCA) was performed for each of the four groups of environmental variables (energy, water, habitat heterogeneity, and human activity).

#### 2.3.4 Geographically weighted regression

Considering the spatial location change of the regression coefficient, geographical coordinates are included as parts of the regression parameters. We performed spatial autocorrelation tests on variables in ArcGIS 10.6 for the analysis of geographical weighted regression (GWR). GWR is an effective method for determining spatially variable parameters by taking into account local‐scale features of geographical elements. The GWR, as proposed by Brunsdon et al. (Brunsdon et al., 1996), is computed as:

$$y_{i}=\sum_{k}\beta_{k}\left(u_{i},v_{i}\right)x_{k,i}+\varepsilon_{i}$$

We used Akaike's Information Criterion (AIC) (Akaike, 1974), which takes into account model complexity and goodness‐of‐fit, to evaluate the OLS and GWR models. A more desirable model is indicated by a smaller AIC.

## 3 RESULTS

Our extensive field survey, which lasted from January 2016 to October 2020, covered all county‐level administrative units (15/15), 91.47% of the elevational range (2370 m/2591 m), and most types of natural landforms and vegetations across the study region. Altogether 260 transects were established, with 2493 quadrats with orchid occurrence set and recorded.

These field‐collected data, in combination with our extensive literature review, documented 249 native orchid species belonging to 74 genera in the Beipan River Basin (Extended Data Table S1). These account for 14.5% and 41.9% of the species and genera of native orchids recorded in China to date, despite a relatively small spatial extent (0.2%) of this area.

### 3.1 Distribution characteristics of species richness and environmental factors

**FIGURE 3:**
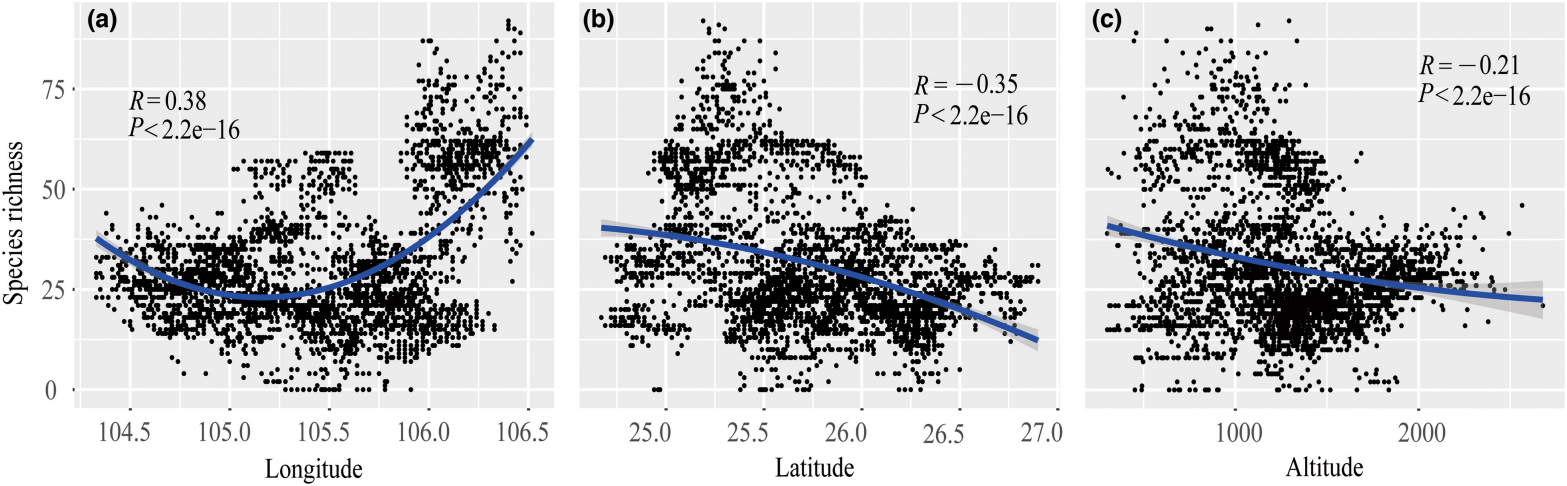
Change in orchid species richness along the longitudinal, latitudinal, and elevational gradients within Beipan River Basin, China, with each dot denoting a grid cell

Consistent with the remarkable environmental heterogeneity (Table 1), the spatial distribution of native orchids is also highly uneven across our study area (Figure 3). There is an extremely significant quadratic correlation (*p* < .001) between orchid species richness and each of longitude, latitude, and elevation.

**TABLE 1:** Correlation analysis of environmental variables and geographical factors in Beipan River basin of Guizhou Province, China

Environmental variable	Longitude (E)	Latitude (N)	Elevation (m)
Energy factors			
MAT, °C	0.59 (UD)***	−0.7 (DD)***	−0.94 (DD)***
MDR, °C	−0.32 (DD)***	−0.22 (DD)***	−0.14 (DU)***
ISO	−0.65 (DU)***	−0.29 (DD)***	0.19 (DU)***
TS, °C	0.76 (UD)***	0.22 (UU)***	−0.4 (UD)***
MTWM, °C	0.71 (UD)***	−0.6 (DD)***	−0.96 (DD)***
MTCM, °C	0.43 (UD)***	−0.8 (DD)***	−0.84 (DU)***
TAR, °C	0.7 (UD)***	0.24 (UU)***	−0.44 (UD)***
MTWetQ, °C	0.68 (UD)***	−0.62 (DD)***	−0.96 (DD)***
MTDQ, °C	0.43 (UD)***	−0.76 (DD)***	−0.87 (DU)***
MTCQ, °C	0.74 (UD)***	−0.58 (DD)***	−0.97 (DD)***
MTWarmQ, °C	0.43 (UD)***	−0.76 (DD)***	−0.87 (DU)***
WD, mm	−0.35 (DU)***	0.2 (DU)***	−0.06 (DU)**
PET, mm	0.07 (UD)***	−0.73 (DD)***	−0.62 (DU)***
Water factors			
MAP, mm	0.37 (UD)***	−0.82 (UD)***	−0.49 (UD)***
PWM, mm	0.04 (UD)*	−0.7 (UD)***	−0.33 (UD)***
PDM, mm	−0.22 (UD)***	0.43 (UD)***	0.33 (UD)***
PS, mm	−0.53 (DU)***	−0.46 (DU)***	0.13 (DU)***
PWetQ, mm	0.1 (UD)***	−0.9 (DD)***	−0.38 (DD)***
PDQ, mm	0.43 (UD)***	−0.33 (UD)***	−0.38 (UD)***
PWarmQ, mm	0.082 (UD)***	−0.9 (DD)***	−0.37 (DD)***
PCQ, mm	0.43 (UD)***	−0.33 (UD)***	−0.38 (UD)***
MI	0.35 (UD)***	−0.16 (UD)***	0.08 (UD)***
AET, mm	0.80 (UU)***	−0.50 (DD)***	−0.87 (DD)***
Habitat heterogeneity			
ER, m	−0.17 (DU)***	0.03 (DU)	−0.09 (DU)***
NVF	0.01 (DU)	−0.1 (DD)***	−0.07 (DU)***
RMAT, °C	−0.28 (DD)***	0.14 (UU)***	0.05 (DU)**
RMAP, mm	−0.26 (DU)***	−0.08 (UD)***	0.00 (DU)
Human activities			
HPD	−0.09 (DD)***	0.17 (UU)***	0.11 (UD)***
AOC, m^2^	−0.25 (UD)***	0.33 (UD)***	0.21 (UD)***
GDP	−0.44 (DU)***	0.49 (UD)***	0.52 (UU)***

Abbreviations: DD, monotonically decreasing; DU, Conic relation of first decreasing and then increasing; UD, Conic relation of first increase and then decrease; UU, monotonically increasing.

*** *p* < .001.

** *p* < .01.

* *p* < .05.

### 3.2 Influencing factors of species richness pattern and its spatial variation

#### 3.2.1 Determinants of the distribution of orchid species

**FIGURE 4:**
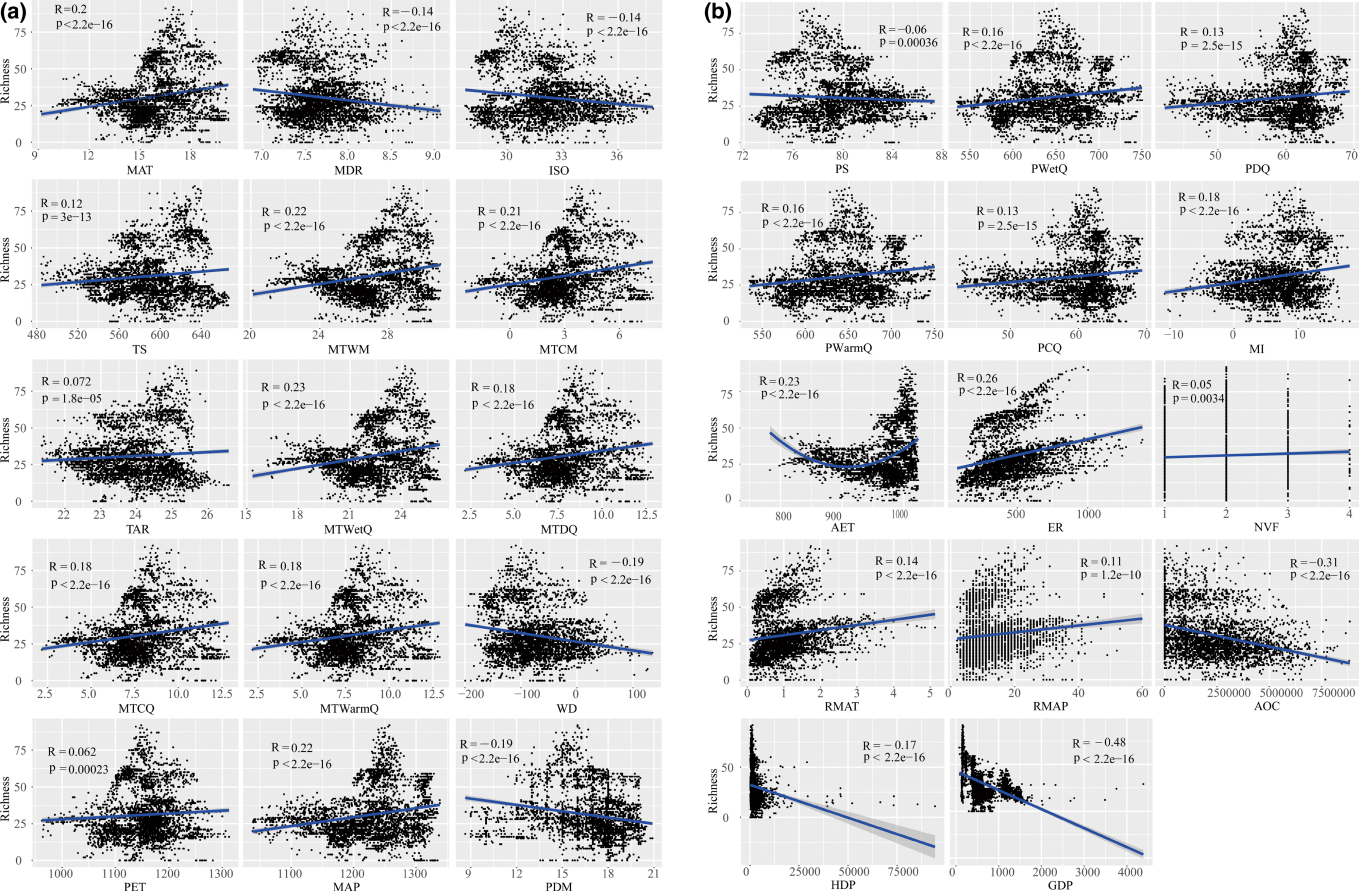
(a) Relationship between wild Orchidaceae species richness and environmental variables in Beipan River basin of Guizhou Province, China. (b) Relationship between wild Orchidaceae species richness and environmental variables in Beipan River basin of Guizhou Province, China

Univariate linear regression was employed to analyze the relationship between each environmental variable and orchid species richness. A significant linear correlation (*p* < .05) was detected between orchid species richness in the Beipan River Basin and all environmental variables but PWM (Figure 4a,b). A total of 153 highly significant (*p* < .001) environmental variables were retained after forward selection, including 131 spatial variations, 10 energy variables (MAT, MDR, ISO, TS, MTWM, MTCM, MTWetQ, MTDQ, MTCQ, and WD), 8 significant water variables (MAP, PDM, PS, PWetQ, PDQ, PWarmQ, MI, and AET), 2 habitat heterogeneity‐related variables (ER and RMAP), and 2 human activity‐related variables (GDP and AOC) (Extended Data Table S2).

**FIGURE 5:**
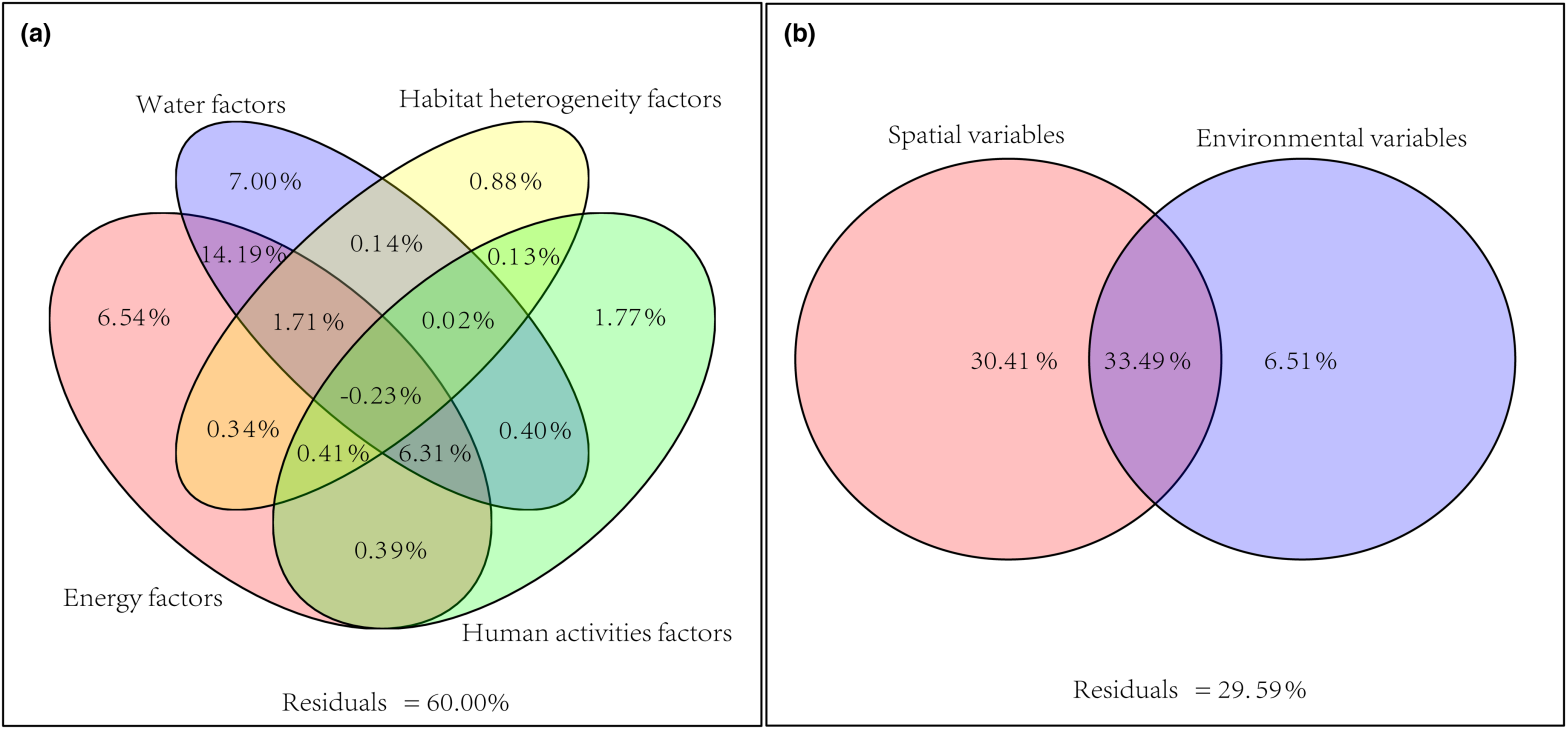
Partition of spatial variance in the distribution of native orchid species in the Beipan River Basin by different variables and their interactions

According to RDA (Figure 5), 40.00% variation in the spatial distribution of native orchid species in the Beipan River Basin can be explained by environmental variables. Proportions of spatial variation explained by each group of environmental variables separately or in interactions with others were detailed in Figure 4a.

Four significant environmental variables, when in combination with spatial variables, jointly accounted for 70.41% variation in the distribution of native orchid species in the Beipan River Basin. The proportions of variation explained by them separately and their interaction are presented in Figure 4b. The results indicated that both the spatial structure of species richness (variation explained solely by spatial variables) and the spatial structure of environmental variables (variation explained by the interaction of spatial and environmental variables) have huge impacts on the spatial distribution of orchid species in our study area. Therefore, the GWR model was employed to analyze the effect of environmental variables on the distribution of orchid species richness at finer spatial scales.

#### 3.2.2 Multicollinearity issues and principal component analysis among environmental variables

**FIGURE 6:**
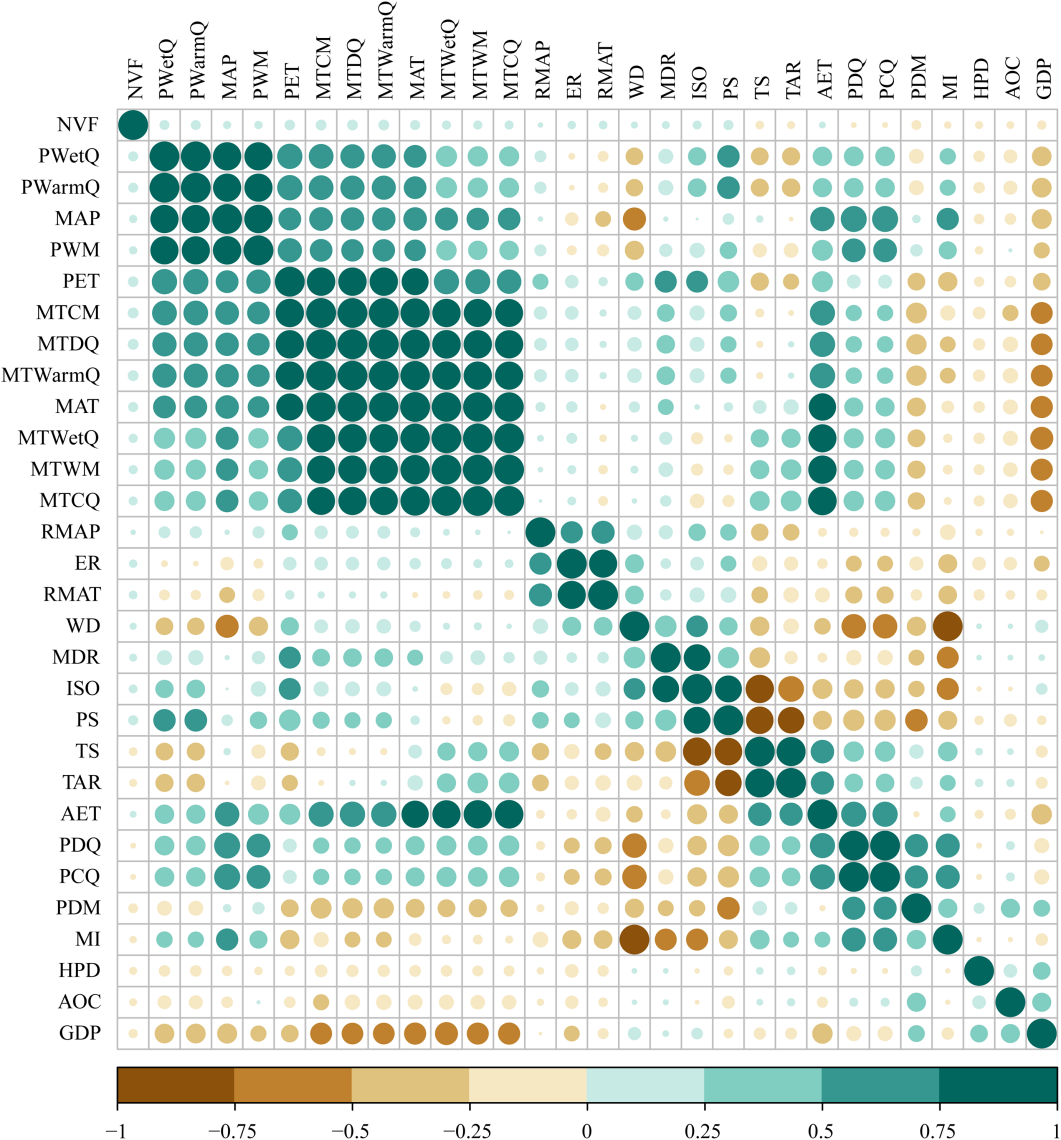
Pearson correlation among environmental factors

Because the correlation between multiple environmental factors was above 0.8, and the significance level was extremely significant (*p* < .001) (Figure 6). Principal component analysis of environmental factors showed that the cumulative proportions of original variance retained by the first two axes (hereafter “principal environmental variables”) were 87.52% for energy variables, 83.67% for water variables, 83.68% for habitat heterogeneity‐related variables, and 80.72% for human activity‐related variables, respectively (Table 2). The scores of these eight principal axes served as candidate variables for performing GWRs below.

**TABLE 2:** Summary of principal component analysis for four groups of environmental variables

Group of environmental variables	Principal component^a^	Eigenvalue	Proportion of variance retained (%)	Cumulative proportion of variance retained (%)
Energy	NL1	7.56	58.15	58.15
	NL2	3.82	29.37	87.52
	NL3	0.97	7.48	95.00
	NL4	0.56	4.30	99.30
	NL5	0.07	0.52	99.82
	NL6	0.02	0.12	99.93
	NL7	0.01	0.04	99.98
	NL8	0.00	0.02	100.00
	NL9	0.00	0.00	100.00
	NL10	0.00	0.00	100.00
	NL11	0.00	0.00	100.00
	NL12	0.00	0.00	100.00
	NL13	0.00	0.00	100.00
Water	SF1	5.41	54.14	54.14
	SF2	2.95	29.53	83.67
	SF3	0.94	9.43	93.11
	SF4	0.58	5.79	98.90
	SF5	0.08	0.81	99.71
	SF6	0.02	0.18	99.89
	SF7	0.01	0.09	99.98
	SF8	0.00	0.01	99.99
	SF9	0.00	0.01	100.00
	SF10	0.00	0.00	100.00
Habitat heterogeneity	SJ1	2.35	58.77	58.77
	SJ2	1.00	24.92	83.68
	SJ3	0.54	13.51	97.19
	SJ4	0.11	2.81	100.00
Human activities	RL1	1.60	53.37	53.37
	RL2	0.82	27.35	80.72
	RL3	0.58	19.28	100.00

*Note*: Since >80% of original variations is retained by the first two axes for each of the four groups, we use them (eight principal components in total) as candidate explanatory variables for geographical weighted regressions, so as to preserve as much original variation as possible, while avoiding multicollinearity issues.

^a^ Principal components are listed by the proportion of original variations they retain.

#### 3.2.3 Model selection

The Moran's I index of global autocorrelation analysis of species richness was 0.85, which passed the test at the significance level of 0.01, showing positive spatial autocorrelation and aggregation distribution. In addition, the z‐value was 70.70, far higher than the critical value (2.58) under the confidence level, indicating that the species richness of wild orchids in the Beipan River Basin of Guizhou province had a significant positive spatial autocorrelation. Therefore, it can be analyzed by the geographical weighted regression model.

The coefficient of ordinary least square (OLS) regression indicates the strength and mode of the relationship between environmental variables and the response variable. With OLS, we detected a negative correlation between orchid species richness and principal environmental variables of NL1, SJ2, and RL1, while a positive correlation between orchid species richness and five others (Table 3). Since two of these correlations (those pertaining to SF2 and SJ2) were nonsignificant with *p* > .05, we excluded them and used the remaining six principal environmental variables to perform GWR. All variance inflation factors (VIF) associated with these remaining ones fell below the threshold of 7.5, indicating negligible issues of multicollinearity.

**TABLE 3:** Variance inflation factors for each of the eight (4 × 2) PCA axes and summary of ordinary least square regression

Variables	Coefficient	*p*	VIF
Energy factor 1 (NL1)	−.501	.000	1.866
Energy factor 2 (NL2)	1.664	.000	2.971
Water factor 1 (SF1)	.830	.000	1.544
Water factor 2 (SF2)	.327	.189	3.194
Habitat heterogeneity factor 1 (SJ1)	2.314	.000	1.243
Habitat heterogeneity factor 2 (SJ2)	−.192	.428	1.012
Human activities factor 1 (RL1)	−5.110	.000	1.421
Human activities factor 2 (RL2)	2.028	.000	1.046

The GWR model was able to explain 70.08% of the variance of spatial distribution of orchid species richness in the Beipan River Basin, indicating its marked superiority over the OLS model, which explained only 26.81% (Table 4). Such relative advantage of GWR is also corroborated by other goodness‐of‐fit indices such as adjusted *R* ^2^ and values of small sample‐corrected Akaike Information Criterion (AICc). We therefore believe that GWR is a considerably more effective approach for analyzing the role of environmental variables in shaping the spatial distribution of orchid species in our study region.

**TABLE 4:** Comparison on the performance of ordinary least square (OLS) and geographical weighted regression (GWR)

Model	*R* ^2^	Adjusted *R* ^2^	AICc
OLS	.270	.268	28494.583
GWR	.708	.701	25394.420

#### 3.2.4 Effects of principal environmental variables

**FIGURE 7:**
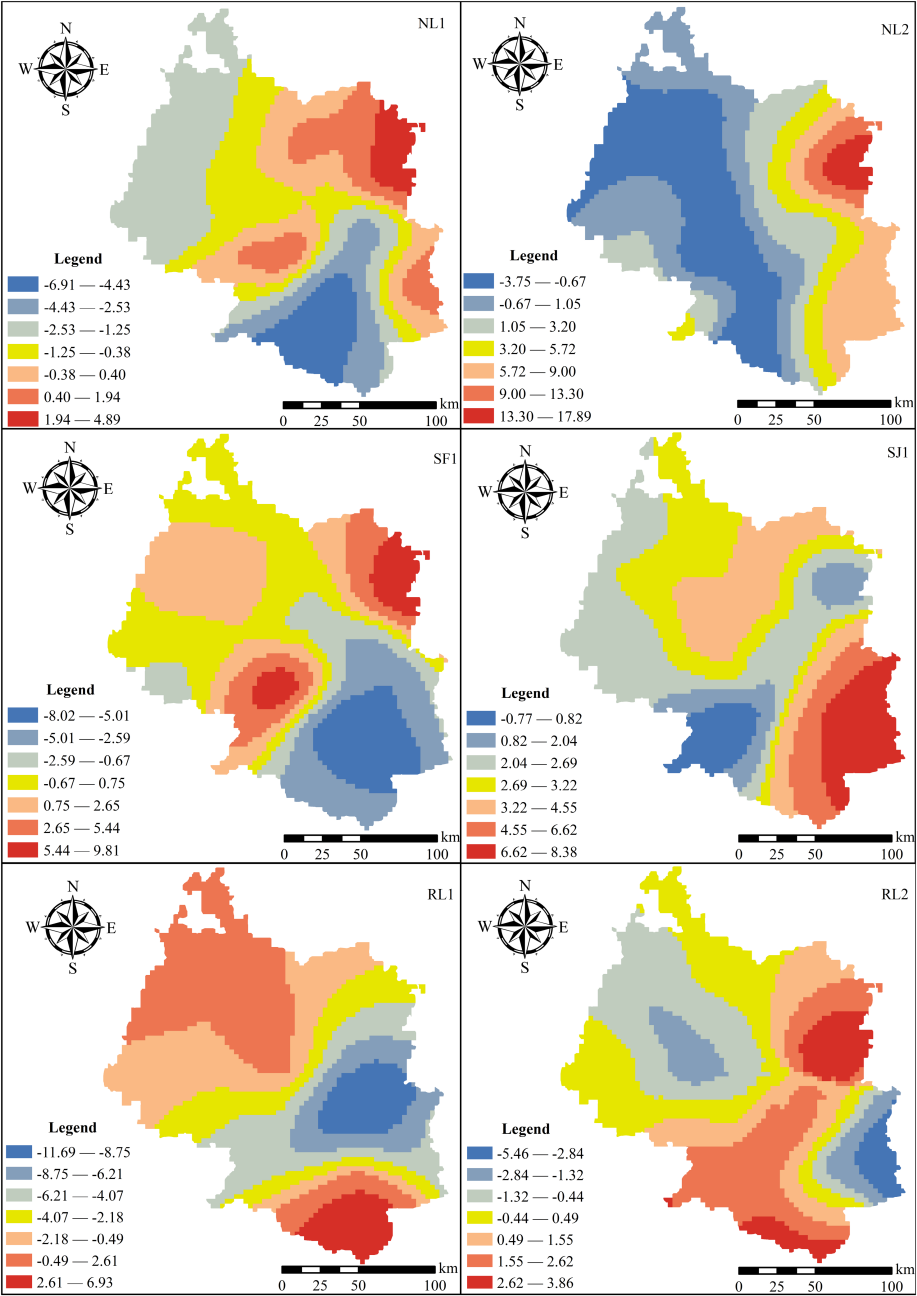
Coefficients of geographically weighted regressions of different environmental variables on spatial distribution of orchid species richness

According to the results of GWR (Figure 7), the effects of the six principal environmental variables on the species richness of native orchids in the Beipan River Basin all displayed substantial spatial heterogeneity and even produced opposite effects across the study area. The effect of SJ1 on orchid species richness was dominated by the number of stimulatory grids (97.26%), on the contrary, NL1 (73.91%) and RL1 (74.96%) showed the predominance of inhibitory grids. In addition, NL2 (57.65%), SF1 (53.39%), and RL2 (50.56%) showed that the grid numbers of facilitation and inhibition were relatively close.

## 4 DISCUSSION

There is significant spatial heterogeneity in the distribution of species richness, which varies with the longitude, latitude, or altitude span of the study area (Rahbek, 2005). Studies have shown that the importance of water, relative to energy, in determining terrestrial plant diversity, varies along the latitudinal gradient. Water plays a primary role in low‐latitude regions, while energy is the more dominant one affecting the geographical pattern of species richness in high latitudes (Eiserhardt et al., 2011; Kreft & Jetz, 2007; Oliveira & Diniz‐Filho, 2010; Xu et al., 2013). In this study, the species richness of orchids first decreased and then increased with longitude, which can be explained by the degraded habitat quality, sore desertification, cattle grazing, and anthropogenic excavation prevailing in the central part of our study area (Chen et al., 2016; Peng et al., 2013). It shows a monotonous decreasing trend with increasing latitude and altitude, which may be related to the downstream area of Beipan River in Guizhou province is located mainly in lower latitudes, with lower elevation accompanied by stretching mountains and desirable humidity and heat, allowing native orchid flora to thrive.

Spatial variables were able to account for 63.90% of the spatial variation of orchid distribution, much higher than the proportion explained by environmental variables (40.00%). This indicates that spatial structure played an important role in shaping the spatial distribution of orchid species richness in Beipan River of Guizhou province. Consistent with Gravel et al. (2006), who argue that niche differentiation plays a dominant role in the distribution of biodiversity (implying a weakened role of the spatial structure of the environment) in areas with less fertile sediments and/or with simplified species composition, karst landforms in Beipan River Basin are highly developed with abundant rainfall, complex habitat structures, and diverse biotic assemblies. It is therefore comprehensible that spatial structure plays a dominant role in the distribution of species richness of wild orchids in our study area. The interaction between environmental variables and spatial variables explained 33.49% of total spatial variations, indicating that the spatial structure of environmental variables at least partly contributed to the spatial heterogeneity of orchid species richness (Borcard et al., 2018). The proportion of spatial variation explained solely by spatial variables was 30.41%, which is often deemed to derive mainly from the spatial autocorrelation of species distribution (La et al., 2016). Nonetheless, this part of the variation in orchid distribution may also arise from historical geological events (Svenning & Skov, 2010), diffusion limitations (Gilbert & Lechowicz, 2004), or other unidentified environmental factors (Smith & Lundholm, 2010).

Despite that an array of environmental variables have been selected, 29.59% variance of the spatial distribution of native orchid flora in our study area remained unexplained. This may be due to the lack of information about the symbiotic fungi which orchids rely on to thrive. The occurrence of orchids is generally sensitive to the ambient environment, and texture, water content, as well as physical and chemical properties of the sediments, are all among the critical factors determining the existence of orchid flora (Li et al., 2009). In addition, all orchids known to date must rely on symbiotic fungi for nutrition at least during their early life stages (Arditti & Ghani, 2000). Therefore, soil properties and the presence of eligible microorganisms may as well serve as major factors affecting the spatial pattern of species richness of wild orchids in Beipan River Basin of China. In order to bridge such gaps, we here call for future studies to incorporate field‐collected environmental data (particularly those difficult to obtain online, such as the abundance of selected symbiotic fungi [McCormick et al., 2018]) in explaining orchid distribution, so as to facilitate more detailed and in‐depth scientific understanding on the limiting environmental factors of these fragile, charming plants, especially given the dire threats of projected climate change.

There is significant spatial heterogeneity in each environmental factor affecting the species richness of orchids. Although there is abundant rainfall in the study area, the karst landforms are well‐developed and their ability to retain water is weak. Therefore, the effect of water factor 1 (SF1) on species richness of orchids is dominant in the promoting grid number, while that of SF1 was dominant in the inhibiting grid number in the non‐karst region. However, the effect of habitat heterogeneity factor 1 (SJ1) on the species richness of orchids in the study area is dominated by the number of grids that promote the effect, that is, there is a significant positive correlation between SJ1 and orchid richness. Therefore, the diversity of orchids within a certain area was argued to serve, to some extent, as a proxy of overall biodiversity (Jin et al., 2011).

## 5 CONCLUSION

The area of the Beipan River basin in Guizhou accounts for about 11.88% of the land area of Guizhou Province, but 80.43% of the genera and 72.59% of the species of wild orchids in Guizhou Province are distributed. It is one of the most abundant areas of wild orchids in Guizhou Province. In general, the species richness of wild orchids in Beipan River showed a pattern of “high in southeast and low in northwest, and east‐west differentiation,” which was influenced by environmental factors, spatial structure of environmental factors, and spatial structure of species richness. Our research provides detailed geographical distribution of wild orchids, diversity enrichment area information, and the influence of human activities on the distribution of orchid species richness in the Beipan River Basin of Guizhou Province, provides basic data for the management department to formulate scientific conservation management strategies, and efficiently carries out in situ and ex situ of orchids. It provides a certain reference for the site selection of conservationists to carry out orchid conservation activities. At the same time, it also provides a good example for species conservation and exploring the drivers of distributed patterns in other areas with rich orchid diversity in future. In order to strengthen the conservation of orchids in the Beipan River Basin of Guizhou Province, we suggest that in situ should be carried out in the areas where orchids are relatively concentrated, and appropriate ex situ should be carried out that are greatly affected by human activities. At the same time, relevant competent departments should strengthen the publicity of orchid conservation, and increase the punishment for destroying orchid habitats and overmining orchids. Fortunately, under the leadership of relevant departments, a conservation base for orchids has been built in the region, and the population monitoring of some rare and endangered orchids has been carried out in succession.

## AUTHOR CONTRIBUTIONS

**chao Ye:** Conceptualization (lead); data curation (lead); formal analysis (lead); investigation (lead); methodology (lead); project administration (supporting); resources (supporting); software (lead); supervision (lead); validation (lead); visualization (lead); writing – original draft (lead); writing – review and editing (lead). **mingtai an:** Conceptualization (equal); data curation (equal); formal analysis (equal); funding acquisition (lead); investigation (equal); methodology (equal); project administration (equal); resources (equal); software (equal); supervision (equal); validation (equal); visualization (equal); writing – original draft (equal); writing – review and editing (equal). **jinzhu shi:** Data curation (supporting); formal analysis (supporting); investigation (equal); methodology (supporting); supervision (supporting); validation (supporting); visualization (supporting); writing – review and editing (supporting). **feng liu:** Data curation (supporting); formal analysis (supporting); investigation (equal); methodology (supporting); supervision (supporting); validation (supporting); visualization (supporting); writing – review and editing (supporting). **yang zhang:** Data curation (supporting); formal analysis (supporting); investigation (equal); methodology (supporting); supervision (supporting); validation (supporting); visualization (supporting); writing – review and editing (supporting).

## CONFLICT OF INTEREST

The authors declare that they have no conflict of interest.

## Supporting information


Appendix S1

## Data Availability

Data associated with this manuscript can be accessed at the Dryad data repository (https://doi.org/10.5061/dryad.k0p2ngfbx).
