# Supplementary material for: Spatial distribution and its limiting environmental factors of native orchid species diversity in the Beipan River Basin of Guizhou Province, China
Source: Ecol Evol. 2022 Nov 14;12(11):e9470. doi: 10.1002/ece3.9470 (PMC9663322; doi:10.1002/ece3.9470)
Supplement: Supplementary file 1 — Appendix S1 [file ECE3-12-e9470-s001.docx]

# Appendix

**Extended Data Tab. 1 A list of orchids in the Beipan River Basin of Guizhou Province**

| **Genus** | **Latin name** | **CITES** | **IUCN red list categories** |
| --- | --- | --- | --- |
| *Acampe* | *Acampe* *rigida* (Buch.-Ham. ex Sm.) P.F.Hunt | Ⅱ |  |
| *Aerides* | *Aerides rosea* Loddiges ex Lindl. & Paxton | Ⅱ |  |
| *Anoectochilus* | *Anoectochilus* *roxburghii* (Wall.) Lindl. | Ⅱ |  |
| *Anoectochilus* | *Anoectochilus* *xingrenensis* Z.H.Tsi & X.H.Jin | Ⅱ |  |
| *Anthogonium* | *Anthogonium* *gracile* Lindl. | Ⅱ |  |
| *Aphyllorchis* | *Aphyllorchis* *gollanii* Duthie | Ⅱ |  |
| *Aphyllorchis* | *Aphyllorchis* *montana* Rchb.f. | Ⅱ |  |
| *Arundina* | *Arundina* *graminifolia* (D.Don) Hochreutiner | Ⅱ |  |
| *Bletilla* | *Bletilla* *formosana* (Hayata) Schltr. | Ⅱ |  |
| *Bletilla* | *Bletilla* *ochracea* Schltr. | Ⅱ |  |
| *Bletilla* | *Bletilla* *striata* (Thunb.) Rchb.f. | Ⅱ |  |
| *Brachycorythis* | *Brachycorythis* *galeandra* (Rchb.f.) | Ⅱ |  |
| *Brachycorythis* | *Brachycorythis* *henryi* (Schltr.) Summerhayes | Ⅱ |  |
| *Bulbophyllum* | *Bulbophyllum* *amplifolium* (Rolfe) N.P.Balakrishnan & Sud.Chowdhury | Ⅱ |  |
| *Bulbophyllum* | *Bulbophyllum* *andersonii* (Hook.f.) J.J.Sm. | Ⅱ |  |
| *Bulbophyllum* | *Bulbophyllum* *delitescens* Hance | Ⅱ | LC |
| *Bulbophyllum* | *Bulbophyllum* *drymoglossum* Maxim. ex Okubo | Ⅱ |  |
| *Bulbophyllum* | *Bulbophyllum* *funingense* Z.H.Tsi & S.C.Chen | Ⅱ |  |
| *Bulbophyllum* | *Bulbophyllum* *kwangtungense* Schltr. | Ⅱ |  |
| *Bulbophyllum* | *Bulbophyllum* *odoratissimum* (Sm.) Lindl. | Ⅱ |  |
| *Bulbophyllum* | *Bulbophyllum* *reptans* (Lindl.) Lindl. | Ⅱ |  |
| *Bulbophyllum* | *Bulbophyllum* *retusiusculum* Rchb.f. | Ⅱ |  |
| *Bulbophyllum* | *Bulbophyllum* *stenobulbon* E.C.Parish & Rchb.f. | Ⅱ |  |
| *Bulbophyllum* | *Bulbophyllum* *tianguii* K.Y.Lang & D.Luo | Ⅱ |  |
| *Bulbophyllum* | *Bulbophyllum* *violaceolabellum* Seidenf. | Ⅱ |  |
| *Calanthe* | *Calanthe* *alismatifolia* Lindl. | Ⅱ |  |
| *Calanthe* | *Calanthe* *arcuata* Rolfe | Ⅱ |  |
| *Calanthe* | *Calanthe* *argenteostriata* C.Z.Tang & S.J.Cheng | Ⅱ |  |
| *Calanthe* | *Calanthe* *davidii* Franchet | Ⅱ |  |
| *Calanthe* | *Calanthe* *discolor* Lindl. | Ⅱ |  |
| *Calanthe* | *Calanthe* *fargesii* Finet | Ⅱ | Vu |
| *Calanthe* | *Calanthe* *graciliflora* Hayata | Ⅱ |  |
| *Calanthe* | *Calanthe* *henryi* Rolfe | Ⅱ | Vu |
| *Calanthe* | *Calanthe* *mannii* Hook.f. | Ⅱ |  |
| *Calanthe* | *Calanthe* *odora* Griff. | Ⅱ |  |
| *Calanthe* | *Calanthe* *puberula* Lindl. | Ⅱ |  |
| *Calanthe* | *Calanthe* *reflexa* Maxim. | Ⅱ |  |
| *Calanthe* | *Calanthe* *tricarinata* Lindl. | Ⅱ |  |
| *Calanthe* | *Calanthe* *triplicata* (Willemet) Ames | Ⅱ |  |
| *Calanthe* | *Calanthe* *tsoongiana* Tang & F.T.Wang | Ⅱ |  |
| *Calanthe* | *Calanthe* *wuxiensis* H.P.Deng & F.Q.Yu | Ⅱ |  |
| *Cephalanthera* | *Cephalanthera* *damasonium* (Miller) Druce | Ⅱ |  |
| *Cephalanthera* | *Cephalanthera* *erecta* (Thunb.) Bl. | Ⅱ |  |
| *Cephalanthera* | *Cephalanthera* *falcata* (Thunb.) Bl. | Ⅱ |  |
| *Cephalanthera* | *Cephalanthera* *longifolia* (L.) Fritsch | Ⅱ |  |
| *Cephalanthera* | *Cephalanthera* *nanlingensis* A.Q.Hu & F.W.Xing | Ⅱ |  |
| *Chamaegastrodia* | *Chamaegastrodia* *inverta* (W.W.Sm.) Seidenf. | Ⅱ |  |
| *Cheirostylis* | *Cheirostylis* *chinensis* Rolfe | Ⅱ |  |
| *Cheirostylis* | *Cheirostylis* *malipoensis* X.H.Jin & S.C.Chen | Ⅱ |  |
| *Cheirostylis* | *Cheirostylis* *yunnanensis* Rolfe | Ⅱ |  |
| *Chiloschista* | *Chiloschista* *yunnanensis* Schltr. | Ⅱ |  |
| *Cleisostoma* | *Cleisostoma* *fuerstenbergianum* Kraenz. | Ⅱ |  |
| *Cleisostoma* | *Cleisostoma* *menghaiense* Z.H.Tsi | Ⅱ |  |
| *Cleisostoma* | *Cleisostoma* *nangongense* Z.H.Tsi | Ⅱ |  |
| *Cleisostoma* | *Cleisostoma* *paniculatum* (Ker Gawler) Garay | Ⅱ |  |
| *Cleisostoma* | *Cleisostoma* *parishii* (Hook.f.) Garay | Ⅱ |  |
| *Cleisostoma* | *Cleisostoma* *rostratum* (Loddiges ex Lindl.) Garay | Ⅱ |  |
| *Cleisostoma* | *Cleisostoma* *williamsonii* (Rchb.f.) Garay | Ⅱ |  |
| *Coelogyne* | *Coelogyne* *corymbosa* Lindl. | Ⅱ |  |
| *Coelogyne* | *Coelogyne* *fimbriata* Lindl. | Ⅱ |  |
| *Coelogyne* | *Coelogyne* *flaccida* Lindl. | Ⅱ |  |
| *Collabium* | *Collabium* *formosanum* Hayata | Ⅱ |  |
| *Conchidium* | *Conchidium* *rhomboidale* (Tang & F.T.Wang) S.C.Chen & J.J.Wood | Ⅱ |  |
| *Corallorhiza* | *Corallorhiza* *trifida* Châtelain | Ⅱ |  |
| *Cremastra* | *Cremastra* *appendiculata* (D.Don) Makino | Ⅱ |  |
| *Cremastra* | *Cremastra* *guizhouensis* Q.H.Chen & S.C.Chen | Ⅱ |  |
| *Crepidium* | *Crepidium* *acuminatum* (D.Don) Szlach. | Ⅱ |  |
| *Crepidium* | *Crepidium* *biauritum* (Lindl.) Szlach. | Ⅱ |  |
| *Crepidium* | *Crepidium* *matsudae* (Yamamoto) Szlach. | Ⅱ |  |
| *Crepidium* | *Crepidium* *purpureum* (Lindl.) Szlach. | Ⅱ |  |
| *Cymbidium* | *Cymbidium* *aloifolium* (Linnaeus) Swartz | Ⅱ |  |
| *Cymbidium* | *Cymbidium* *cyperifolium* Wall. ex Lindl. | Ⅱ |  |
| *Cymbidium* | *Cymbidium* *cyperifolium* var. *szechuanicum* (Y. S. Wu et S. C. Chen) S. C. Chen et Z. J. Liu | Ⅱ |  |
| *Cymbidium* | *Cymbidium* *defoliatum* Y.S.Wu & S.C.Chen | Ⅱ | EN |
| *Cymbidium* | *Cymbidium* *ensifolium* (L.) Sw. | Ⅱ |  |
| *Cymbidium* | *Cymbidium* *erythraeum* Lindl. | Ⅱ |  |
| *Cymbidium* | *Cymbidium* *faberi* Rolfe | Ⅱ |  |
| *Cymbidium* | *Cymbidium* *floribundum* Lindl.(?C.chawalongense) | Ⅱ |  |
| *Cymbidium* | *Cymbidium* *goeringii* (Rchb.f.) Rchb.f. | Ⅱ |  |
| *Cymbidium* | *Cymbidium* *hookerianum* Rchb.f. | Ⅱ |  |
| *Cymbidium* | *Cymbidium* *iridioides* D.Don | Ⅱ |  |
| *Cymbidium* | *Cymbidium* *kanran* Makino | Ⅱ |  |
| *Cymbidium* | *Cymbidium* *lancifolium* Hook. | Ⅱ |  |
| *Cymbidium* | *Cymbidium* *macrorhizum* Lindl. | Ⅱ |  |
| *Cymbidium* | *Cymbidium* *mannii* Rchb.f. | Ⅱ |  |
| *Cymbidium* | *Cymbidium* *nanulum* Y.S.Wu & S.C.Chen | Ⅱ | EN |
| *Cymbidium* | *Cymbidium* *qiubeiense* K.M.Feng & H.Li | Ⅱ |  |
| *Cymbidium* | *Cymbidium* *recurvatum* Z.J.Liu, S.C.Chen & P.J.Cribb | Ⅱ |  |
| *Cymbidium* | *Cymbidium* *rhizomatosum* Z.J.Liu & S.C.Chen | Ⅱ |  |
| *Cymbidium* | *Cymbidium* *serratum* Schltr. | Ⅱ |  |
| *Cymbidium* | *Cymbidium* *sinense* (Jack. ex Andr.) Willd. | Ⅱ |  |
| *Cymbidium* | *Cymbidium* *suavissimum* Sander ex C.H.Curtis | Ⅱ |  |
| *Cymbidium* | *Cymbidium* *tortisepalum* var. *longibracteatum* (Y. S. Wu et S. C. Chen) S. C. Chen et Z. J. Liu | Ⅱ |  |
| *Cymbidium* | *Cymbidium* *tracyanum* L.Castle | Ⅱ | LC |
| *Cypripedium* | *Cypripedium* *lichiangense* S.C.Chen & P.J.Cribb | Ⅱ | EN |
| *Cyrtosia* | *Cyrtosia* *nana* (Rolfe ex Downie) Garay | Ⅱ |  |
| *Dendrobium* | *Dendrobium* *aduncum* Wall. ex Lindl. | Ⅱ |  |
| *Dendrobium* | *Dendrobium* *chrysanthum* Wall. ex Lindl. | Ⅱ |  |
| *Dendrobium* | *Dendrobium* *aphyllum* (Roxb.) C.E.C.Fischer | Ⅱ |  |
| *Dendrobium* | *Dendrobium* *denneanum* Kerr | Ⅱ |  |
| *Dendrobium* | *Dendrobium* *fimbriatum* Hook. | Ⅱ |  |
| *Dendrobium* | *Dendrobium* *hancockii* Rolfe | Ⅱ |  |
| *Dendrobium* | *Dendrobium* *henryi* Schltr. | Ⅱ |  |
| *Dendrobium* | *Dendrobium* *hercoglossum* Rchb.f. | Ⅱ |  |
| *Dendrobium* | *Dendrobium* *lindleyi* Steudel | Ⅱ |  |
| *Dendrobium* | *Dendrobium* *loddigesii* Rolfe | Ⅱ |  |
| *Dendrobium* | *Dendrobium* *lohohense* Tang & F.T.Wang | Ⅱ | EN |
| *Dendrobium* | *Dendrobium* *moniliforme* (L.) Swartz | Ⅱ |  |
| *Dendrobium* | *Dendrobium* *nobile* Lindl. | Ⅱ |  |
| *Dendrobium* | *Dendrobium* *officinale* Kimura & Migo | Ⅱ | CR |
| *Dendrobium* | *Dendrobium* *scoriarum* W.W.Sm. | Ⅱ |  |
| *Dendrobium* | *Dendrobium* *sinominutiflorum* S.C.Chen, J.J.Wood & H.P.Wood | Ⅱ | EN |
| *Dendrobium* | *Dendrobium* *strongylanthum* Rchb.f. | Ⅱ |  |
| *Dendrolirium* | *Dendrolirium* *tomentosum* (J.Koenig) S.C.Chen & J.J.Wood | Ⅱ |  |
| *Didymoplexis* | *Didymoplexis* *vietnamica* Ormerod | Ⅱ |  |
| *Dienia* | *Dienia* *ophrydis* (J.Koenig) Ormerod & Seidenf. | Ⅱ |  |
| *Epigeneium* | *Dendrobium* *amplum* Lindl. ex Wall. | Ⅱ |  |
| *Epipactis* | *Epipactis* *helleborine* (L.) Crantz | Ⅱ |  |
| *Epipactis* | *Epipactis* *mairei* Schltr. | Ⅱ |  |
| *Eria* | *Eria* *clausa* King & Pantl. | Ⅱ |  |
| *Eria* | *Eria* *scabrilinguis* Lindl. | Ⅱ |  |
| *Eria* | *Eria* *coronaria* (Lindl.) Rchb.f. | Ⅱ |  |
| *Erythrodes* | *Erythrodes* *blumei* (Lindl.) Schltr. | Ⅱ | LC |
| *Eulophia* | *Eulophia* *dabia* (D.Don) Hochr. | Ⅱ |  |
| *Eulophia* | *Eulophia* *graminea* Lindl. | Ⅱ |  |
| *Eulophia* | *Eulophia* *sooi* Chun & Tang ex S.C.Chen | Ⅱ |  |
| *Eulophia* | *Eulophia* *zollingeri* (Rchb.f.) J.J.Sm. | Ⅱ |  |
| *Flickingeria* | *Flickingeria* *calocephala* Z. H. Tsi et S. C. Chen | Ⅱ |  |
| *Flickingeria* | *Flickingeria* *fimbriata* (Bl.) Hawkes | Ⅱ |  |
| *Flickingeria* | *Flickingeria* *tricarinata* Z. H. Tsi et S. C. Chen | Ⅱ |  |
| *Galeola* | *Galeola* *faberi* Rolfe | Ⅱ |  |
| *Galeola* | *Galeola* *lindleyana* (Hook.f. & Thomson) Rchb.f. | Ⅱ |  |
| *Gastrochilus* | *Gastrochilus* *minutiflorus* Aver. | Ⅱ |  |
| *Gastrodia* | *Gastrodia* *elata* Bl. | Ⅱ | Vu |
| *Geodorum* | *Geodorum* *densiflorum* (Lam.) Schltr. | Ⅱ |  |
| *Geodorum* | *Geodorum* *eulophioides* Schltr. | Ⅱ |  |
| *Goodyera* | *Goodyera* *biflora* (Lindl.) Hook.f. | Ⅱ |  |
| *Goodyera* | *Goodyera* *bomiensis* K.Y.Lang | Ⅱ |  |
| *Goodyera* | *Goodyera* *brachystegia* Hand.-Mazz. | Ⅱ |  |
| *Goodyera* | *Goodyera* *kwangtungensis* C.L.Tso | Ⅱ |  |
| *Goodyera* | *Goodyera* *procera* (Ker Gawler) Hooker | Ⅱ |  |
| *Goodyera* | *Goodyera* *repens* (L.) R.Br. | Ⅱ |  |
| *Goodyera* | *Goodyera* *robusta* Hook.f. | Ⅱ |  |
| *Goodyera* | *Goodyera* *rubicunda* (Bl.) Lindl. | Ⅱ |  |
| *Goodyera* | *Goodyera* *schlechtendaliana* Rchb.f. | Ⅱ |  |
| *Goodyera* | *Goodyera* *viridiflora* (Bl.) Lindl. ex D.Dietrich | Ⅱ |  |
| *Habenaria* | *Habenaria* *aitchisonii* Rchb.f. | Ⅱ |  |
| *Habenaria* | *Habenaria* *ciliolaris* Kraenzl. | Ⅱ |  |
| *Habenaria* | *Habenaria* *davidii* Franch. | Ⅱ |  |
| *Habenaria* | *Habenaria* *delavayi* Finet | Ⅱ | Vu |
| *Habenaria* | *Habenaria* *dentata* (Sw.) Schltr. | Ⅱ |  |
| *Habenaria* | *Habenaria* *fordii* Rolfe | Ⅱ | Vu |
| *Habenaria* | *Habenaria* *furcifera* Lindl. | Ⅱ |  |
| *Habenaria* | *Habenaria* *glaucifolia* Bureau & Franch. | Ⅱ |  |
| *Habenaria* | *Habenaria* *limprichtii* Schltr. | Ⅱ |  |
| *Habenaria* | *Habenaria* *linguella* Lindl. | Ⅱ |  |
| *Habenaria* | *Habenaria* *luquanensis* G. W. Hu | Ⅱ |  |
| *Habenaria* | *Habenaria* *petelotii* Gagnep. | Ⅱ |  |
| *Habenaria* | *Habenaria* *plurifoliata* Tang & F.T.Wang | Ⅱ |  |
| *Habenaria* | *Habenaria* *rostellifera* Rchb.f. | Ⅱ |  |
| *Habenaria* | *Habenaria* *rostrata* Lindl. | Ⅱ |  |
| *Habenaria* | *Habenaria* *shweliensis* W.W.Sm. & Banerji | Ⅱ |  |
| *Habenaria* | *Habenaria* *siamensis* Schltr. | Ⅱ | Vu |
| *Hemipilia* | *Hemipilia* *flabellata* Bureau & Franch. | Ⅱ | Vu |
| *Hemipilia* | *Hemipilia* *limprichtii* Schltr. | Ⅱ | Vu |
| *Herminium* | *Herminium* *alaschanicum* Maxim. | Ⅱ |  |
| *Herminium* | *Herminium* *lanceum* (Thunb. ex Sw.) Vuijk | Ⅱ |  |
| *Ischnogyne* | *Ischnogyne* *mandarinorum* (Kraenzl.) Schltr. | Ⅱ |  |
| *Liparis* | *Liparis* *balansae* Gagnep. | Ⅱ |  |
| *Liparis* | *Liparis* *bootanensis* Griff. | Ⅱ |  |
| *Liparis* | *Liparis* *campylostalix* Rchb.f. | Ⅱ |  |
| *Liparis* | *Liparis* *cathcartii* Hook.f. | Ⅱ |  |
| *Liparis* | *Liparis* *cespitosa* (Lam.) Lindl. | Ⅱ |  |
| *Liparis* | *Liparis* *chapaensis* Gagnep. | Ⅱ |  |
| *Liparis* | *Liparis* *cordifolia* Hook.f. | Ⅱ |  |
| *Liparis* | *Liparis* *distans* C.B.Clarke | Ⅱ |  |
| *Liparis* | *Liparis* *esquirolii* Schltr. | Ⅱ |  |
| *Liparis* | *Liparis* *fargesii* Finet | Ⅱ |  |
| *Liparis* | *Liparis* *nervosa* (Thunb.) Lindl. | Ⅱ |  |
| *Liparis* | *Liparis* *nigra* Seidenf. | Ⅱ |  |
| *Liparis* | *Liparis* *pauliana* Hand.-Mazz. | Ⅱ |  |
| *Liparis* | *Liparis* *stricklandiana* Rchb.f. | Ⅱ |  |
| *Liparis* | *Liparis* *viridiflora* (Bl.) Lindl. | Ⅱ |  |
| *Luisia* | *Luisia* *morsei* Rolfe | Ⅱ |  |
| *Luisia* | *Luisia* *teres* (Thunb.) Bl. | Ⅱ |  |
| *Monomeria* | *Monomeria* *barbata* Lindl. | Ⅱ |  |
| *Nervilia* | *Nervilia* *aragoana* Gaudichaud | Ⅱ |  |
| *Nervilia* | *Nervilia* *mackinnonii* (Duthie) Schltr. | Ⅱ |  |
| *Nervilia* | *Nervilia* *plicata* (Andr.) Schltr. | Ⅱ |  |
| *Oberonia* | *Oberonia* *cavaleriei* Finet | Ⅱ |  |
| *Oberonia* | *Oberonia* *ensiformis* (Sm.) Lindl. | Ⅱ |  |
| *Oberonia* | *Oberonia* *kwangsiensis* Seidenf. | Ⅱ |  |
| *Odontochilus* | *Odontochilus* *elwesii* C.B.Clarke ex Hook.f. | Ⅱ |  |
| *Oreorchis* | *Oreorchis* *patens* (Lindl.) Lindl. | Ⅱ |  |
| *Pachystoma* | *Pachystoma* *pubescens* Bl. | Ⅱ |  |
| *Panisea* | *Panisea* *cavaleriei* Schltr. | Ⅱ |  |
| *Paphiopedilum* | *Paphiopedilum* *armeniacum* S.C.Chen & F.Y.Liu | Ⅰ | EN |
| *Paphiopedilum* | *Paphiopedilum* *barbigerum* Tang & F.T.Wang | Ⅰ | EN |
| *Paphiopedilum* | *Paphiopedilum* *bellatulum* (Rchb.f.) Stein | Ⅰ | EN |
| *Paphiopedilum* | *Paphiopedilum* *concolor* (Lindl. ex Bateman) Pfitz. | Ⅰ | EN |
| *Paphiopedilum* | *Paphiopedilum* *dianthum* Tang & F.T.Wang | Ⅰ | EN |
| *Paphiopedilum* | *Paphiopedilum* *hirsutissimum* (Lindl. ex Hook.) Stein | Ⅰ | Vu |
| *Paphiopedilum* | *Paphiopedilum* *malipoense* S.C.Chen & Z.H.Tsi | Ⅰ | EN |
| *Paphiopedilum* | *Paphiopedilum* *micranthum* Tang & F.T.Wang | Ⅰ | CR |
| *Pecteilis* | *Pecteilis* *susannae* (L.) Raf. | Ⅱ |  |
| *Pelatantheria* | *Pelatantheria* *bicuspidata* Tang & F.T.Wang | Ⅱ |  |
| *Peristylus* | *Peristylus* *affinis* (D.Don) Seidenf. | Ⅱ |  |
| *Peristylus* | *Peristylus* *coeloceras* Finet | Ⅱ |  |
| *Herminium* | *Herminium* *forceps* (Finet) Schltr. | Ⅱ |  |
| *Peristylus* | *Peristylus* *goodyeroides* (D.Don) Lindl. | Ⅱ |  |
| *Phaius* | *Phaius* *columnaris* C.Z.Tang & S.J.Cheng | Ⅱ |  |
| *Phaius* | *Phaius* *flavus* (Bl.) Lindl. | Ⅱ |  |
| *Phaius* | *Phaius* *mishmensis* (Lindl. & Paxton) Rchb.f. | Ⅱ |  |
| *Phaius* | *Phaius* *tancarvilleae* (L’Héritier) Bl. | Ⅱ |  |
| *Phalaenopsis* | *Phalaenopsis* *wilsonii* Rolfe | Ⅱ |  |
| *Pholidota* | *Pholidota* *articulata* Lindl. | Ⅱ |  |
| *Pholidota* | *Pholidota* *cantonensis* Rolfe | Ⅱ |  |
| *Pholidota* | *Pholidota* *chinensis* Lindl. | Ⅱ | NT |
| *Pholidota* | *Pholidota* *leveilleana* Schltr. | Ⅱ |  |
| *Pholidota* | *Pholidota* *longipes* S.C.Chen & Z.H.Tsi | Ⅱ |  |
| *Pholidota* | *Pholidota* *missionariorum* Gagnep. | Ⅱ |  |
| *Pholidota* | *Pholidota* *yunnanensis* Rolfe | Ⅱ |  |
| *Pinalia* | *Pinalia* *spicata* (D.Don) S.C.Chen & J.J.Wood | Ⅱ |  |
| *Pinalia* | *Pinalia* *szetschuanica* (Schltr.) S.C.Chen & J.J.Wood | Ⅱ | LC |
| *Platanthera* | *Platanthera* *bakeriana* (King & Pantl.) Kraenzl. | Ⅱ |  |
| *Platanthera* | *Platanthera* *clavigera* Lindl. | Ⅱ |  |
| *Platanthera* | *Platanthera* *curvata* K.Y.Lang | Ⅱ |  |
| *Platanthera* | *Platanthera* *japonica* (Thunb.) Lindl. | Ⅱ |  |
| *Platanthera* | *Platanthera* *mandarinorum* Rchb.f. | Ⅱ |  |
| *Platanthera* | *Platanthera* *minor* (Miq.) Rchb.f. | Ⅱ |  |
| *Pleione* | *Pleione* *bulbocodioides* (Franch.) Rolfe | Ⅱ |  |
| *Pleione* | *Pleione* *pleionoides* (Kraenzl.) Bream & H.Mohr | Ⅱ | Vu |
| *Pleione* | *Pleione* *yunnanensis* (Rolfe) Rolfe | Ⅱ |  |
| *Ponerorchis* | *Ponerorchis* *hemipilioides* (Finet) X.H.Jin, Schuit. & W.T.Jin | Ⅱ |  |
| *Rhomboda* | *Rhomboda* *abbreviata* (Lindl.) Ormerod | Ⅱ |  |
| *Rhomboda* | *Rhomboda* *moulmeinensis* (E.C.Parish & Rchb.f.) Ormerod | Ⅱ |  |
| *Robiquetia* | *Robiquetia* *succisa* (Lindl.) Seidenf. & Garay | Ⅱ |  |
| *Satyrium* | *Satyrium* *nepalense* D.Don | Ⅱ |  |
| *Satyrium* | *Satyrium* *nepalense* var. *ciliatum* (Lindl.) Hook. f. | Ⅱ |  |
| *Spathoglottis* | *Spathoglottis* *pubescens* Lindl. | Ⅱ |  |
| *Spiranthes* | *Spiranthes* *sinensis* (Persoon) Ames | Ⅱ | LC |
| *Spiranthes* | *Spiranthes* *sunii* Boufford & Wen H.Zhang | Ⅱ |  |
| *Tainia* | *Tainia* *angustifolia* (Lindl.) Benth. et Hook. f. | Ⅱ |  |
| *Thelasis* | *Thelasis* *khasiana* Hook.f. | Ⅱ |  |
| *Thunia* | *Thunia* *alba* (Lindl.) Rchb.f. | Ⅱ |  |
| *Vanda* | *Vanda* *concolor* Bl. | Ⅱ |  |
| *Vandopsis* | *Vandopsis* *gigantea* (Lindl.) Pfitz. | Ⅱ |  |
| *Zeuxine* | *Zeuxine* *affinis* (Lindl.) Benth. ex Hook.f. | Ⅱ |  |
| *Zeuxine* | *Zeuxine strateumatica* (L.) Schltr. | Ⅱ | LC |

**Extended Data Tab. 2 Significant variables after forward selection**

| **code** | **variables** | **AdjR2Cum** | **pvalue** | **code** | **variables** | **AdjR2Cum** | **pvalue** |
| --- | --- | --- | --- | --- | --- | --- | --- |
| **131 significant spatial variables** | | | | | | | |
| 1 | MEM5 | 0.04 | 0.001 | 41 | MEM39 | 0.44 | 0.001 |
| 2 | MEM7 | 0.07 | 0.001 | 42 | MEM37 | 0.44 | 0.001 |
| 3 | MEM4 | 0.10 | 0.001 | 43 | MEM57 | 0.45 | 0.001 |
| 4 | MEM3 | 0.12 | 0.001 | 44 | MEM26 | 0.45 | 0.001 |
| 5 | MEM8 | 0.15 | 0.001 | 45 | MEM40 | 0.45 | 0.001 |
| 6 | MEM10 | 0.18 | 0.001 | 46 | MEM55 | 0.46 | 0.001 |
| 7 | MEM11 | 0.20 | 0.001 | 47 | MEM31 | 0.46 | 0.001 |
| 8 | MEM6 | 0.21 | 0.001 | 48 | MEM64 | 0.46 | 0.001 |
| 9 | MEM12 | 0.23 | 0.001 | 49 | MEM45 | 0.46 | 0.001 |
| 10 | MEM1 | 0.24 | 0.001 | 50 | MEM42 | 0.47 | 0.001 |
| 11 | MEM9 | 0.26 | 0.001 | 51 | MEM34 | 0.47 | 0.001 |
| 12 | MEM17 | 0.27 | 0.001 | 52 | MEM84 | 0.47 | 0.001 |
| 13 | MEM2 | 0.28 | 0.001 | 53 | MEM47 | 0.48 | 0.001 |
| 14 | MEM15 | 0.29 | 0.001 | 54 | MEM58 | 0.48 | 0.001 |
| 15 | MEM19 | 0.30 | 0.001 | 55 | MEM50 | 0.48 | 0.001 |
| 16 | MEM13 | 0.31 | 0.001 | 56 | MEM63 | 0.48 | 0.001 |
| 17 | MEM16 | 0.32 | 0.001 | 57 | MEM49 | 0.49 | 0.001 |
| 18 | MEM22 | 0.33 | 0.001 | 58 | MEM83 | 0.49 | 0.001 |
| 19 | MEM14 | 0.34 | 0.001 | 59 | MEM66 | 0.49 | 0.001 |
| 20 | MEM24 | 0.34 | 0.001 | 60 | MEM51 | 0.49 | 0.001 |
| 21 | MEM27 | 0.35 | 0.001 | 61 | MEM96 | 0.49 | 0.001 |
| 22 | MEM20 | 0.36 | 0.001 | 62 | MEM59 | 0.50 | 0.001 |
| 23 | MEM32 | 0.36 | 0.001 | 63 | MEM112 | 0.50 | 0.001 |
| 24 | MEM18 | 0.37 | 0.001 | 64 | MEM91 | 0.50 | 0.001 |
| 25 | MEM41 | 0.38 | 0.001 | 65 | MEM44 | 0.50 | 0.001 |
| 26 | MEM30 | 0.38 | 0.001 | 66 | MEM111 | 0.50 | 0.001 |
| 27 | MEM52 | 0.39 | 0.001 | 67 | MEM69 | 0.51 | 0.001 |
| 28 | MEM23 | 0.39 | 0.001 | 68 | MEM54 | 0.51 | 0.001 |
| 29 | MEM43 | 0.40 | 0.001 | 69 | MEM56 | 0.51 | 0.001 |
| 30 | MEM21 | 0.40 | 0.001 | 70 | MEM46 | 0.51 | 0.001 |
| 31 | MEM28 | 0.41 | 0.001 | 71 | MEM60 | 0.51 | 0.001 |
| 32 | MEM33 | 0.41 | 0.001 | 72 | MEM103 | 0.52 | 0.001 |
| 33 | MEM38 | 0.41 | 0.001 | 73 | MEM67 | 0.52 | 0.001 |
| 34 | MEM61 | 0.42 | 0.001 | 74 | MEM70 | 0.52 | 0.001 |
| 35 | MEM25 | 0.42 | 0.001 | 75 | MEM36 | 0.52 | 0.001 |
| 36 | MEM238 | 0.42 | 0.001 | 76 | MEM68 | 0.52 | 0.001 |
| 37 | MEM29 | 0.43 | 0.001 | 77 | MEM92 | 0.53 | 0.001 |
| 38 | MEM74 | 0.43 | 0.001 | 78 | MEM138 | 0.53 | 0.001 |
| 39 | MEM53 | 0.43 | 0.001 | 79 | MEM87 | 0.53 | 0.001 |
| 40 | MEM35 | 0.44 | 0.001 | 80 | MEM73 | 0.53 | 0.001 |
| 81 | MEM164 | 0.53 | 0.001 | 107 | MEM99 | 0.57 | 0.001 |
| 82 | MEM78 | 0.53 | 0.001 | 108 | MEM93 | 0.57 | 0.001 |
| 83 | MEM77 | 0.54 | 0.001 | 109 | MEM120 | 0.57 | 0.001 |
| 84 | MEM88 | 0.54 | 0.001 | 110 | MEM176 | 0.57 | 0.001 |
| 85 | MEM48 | 0.54 | 0.001 | 111 | MEM97 | 0.57 | 0.001 |
| 86 | MEM107 | 0.54 | 0.001 | 112 | MEM134 | 0.57 | 0.001 |
| 87 | MEM76 | 0.54 | 0.001 | 113 | MEM65 | 0.58 | 0.001 |
| 88 | MEM109 | 0.54 | 0.001 | 114 | MEM113 | 0.58 | 0.001 |
| 89 | MEM75 | 0.55 | 0.001 | 115 | MEM144 | 0.58 | 0.001 |
| 90 | MEM85 | 0.55 | 0.001 | 116 | MEM126 | 0.58 | 0.001 |
| 91 | MEM79 | 0.55 | 0.001 | 117 | MEM125 | 0.58 | 0.001 |
| 92 | MEM81 | 0.55 | 0.001 | 118 | MEM82 | 0.58 | 0.001 |
| 93 | MEM116 | 0.55 | 0.001 | 119 | MEM182 | 0.58 | 0.001 |
| 94 | MEM98 | 0.55 | 0.001 | 120 | MEM72 | 0.58 | 0.001 |
| 95 | MEM86 | 0.55 | 0.001 | 121 | MEM110 | 0.58 | 0.001 |
| 96 | MEM102 | 0.55 | 0.001 | 122 | MEM179 | 0.59 | 0.001 |
| 97 | MEM62 | 0.56 | 0.001 | 123 | MEM119 | 0.59 | 0.001 |
| 98 | MEM105 | 0.56 | 0.001 | 124 | MEM101 | 0.59 | 0.001 |
| 99 | MEM71 | 0.56 | 0.001 | 125 | MEM95 | 0.59 | 0.001 |
| 100 | MEM124 | 0.56 | 0.001 | 126 | MEM128 | 0.59 | 0.001 |
| 101 | MEM80 | 0.56 | 0.001 | 127 | MEM121 | 0.59 | 0.001 |
| 102 | MEM108 | 0.56 | 0.001 | 128 | MEM170 | 0.59 | 0.001 |
| 103 | MEM90 | 0.56 | 0.001 | 129 | MEM123 | 0.59 | 0.001 |
| 104 | MEM89 | 0.57 | 0.001 | 130 | MEM94 | 0.59 | 0.001 |
| 105 | MEM132 | 0.57 | 0.001 | 131 | MEM131 | 0.59 | 0.001 |
| 106 | MEM156 | 0.57 | 0.001 |  |  |  |  |
| **24 significant environmental variables** | | | | | | | |
| 1 | MTWetQ | 0.13 | 0.001 | 12 | ER | 0.36 | 0.001 |
| 2 | AET | 0.18 | 0.001 | 13 | PWarmQ | 0.37 | 0.001 |
| 3 | PS | 0.22 | 0.001 | 14 | PDQ | 0.37 | 0.001 |
| 4 | GDP | 0.25 | 0.001 | 15 | TS | 0.38 | 0.001 |
| 5 | PDM | 0.28 | 0.001 | 16 | ISO | 0.38 | 0.001 |
| 6 | PWetQ | 0.30 | 0.001 | 17 | MTWM | 0.39 | 0.001 |
| 7 | MTCM | 0.31 | 0.001 | 18 | WD | 0.39 | 0.001 |
| 8 | MAP | 0.32 | 0.001 | 19 | MAT | 0.39 | 0.001 |
| 9 | MTDQ | 0.33 | 0.001 | 20 | RMAP | 0.40 | 0.001 |
| 10 | MI | 0.34 | 0.001 | 21 | MTCQ | 0.40 | 0.001 |
| 11 | MDR | 0.35 | 0.001 | 22 | AOC | 0.40 | 0.001 |
